# Impact of Temperature and UV Irradiation on Dynamics of NO_2_ Sensors Based on ZnO Nanostructures

**DOI:** 10.3390/nano7100312

**Published:** 2017-10-11

**Authors:** Marcin Procek, Agnieszka Stolarczyk, Tadeusz Pustelny

**Affiliations:** 1 Department of Optoelectronics, Silesian University of Technology, 2 Krzywoustego St., 44-100 Gliwice, Poland; Tadeusz.Pustelny@polsl.pl; 2 Department of Physical Chemistry and Technology of Polymers, Silesian University of Technology, 9 Strzody St., 44-100 Gliwice, Poland; Agnieszka.Stolarczyk@polsl.pl

**Keywords:** gas sensors, nanostructures, zinc oxide (ZnO), NO_2_ sensing mechanisms, photoactivation, ultraviolet (UV) activation

## Abstract

The main object of this study is the improvement of the dynamics of NO_2_ sensors based on ZnO nanostructures. Investigations presented in this paper showed that the combination of temperature and ultraviolet (UV) activation of the sensors can significantly decrease the sensor response and regeneration times. In comparison with the single activation method (elevated temperature or UV), these times for 1 ppm of NO_2_ decreased from about 10 min (or more) to less than 40 s. In addition, at the optimal conditions (200 °C and UV), sensors were very stable, were fully scalable (in the range on NO_2_ concentration of 1–20 ppm) and baseline drift was significantly reduced. Furthermore, in this paper, extensive studies of the influence of temperature and carrier gas (nitrogen and air) on NO_2_ sensing properties of the ZnO nanostructures were conducted. The NO_2_ sensing mechanisms of the sensors operating at elevated temperatures and under UV irradiation were also discussed. Our study showed that sensor responses to NO_2_ and response/regeneration times are comparable from sensor to sensor in air and nitrogen conditions, which suggests that the proposed simple technology connected with well-chosen operation conditions is repeatable. The estimated limit of detection of the sensors is within the level of ≈800 ppb in nitrogen and ≈700 ppb in air.

## 1. Introduction

The detection of nitrogen dioxide (NO_2_) is very important due to its high toxicity. NO_2_ sensors are used in the detection of explosive vapors, the automotive industry and the monitoring of atmospheric air [1,2]. Some of these applications require the detection of NO_2_ at low concentrations in the row of parts per billion (ppb) and single parts per million (ppm). The most common materials used for the detection of NO_2_ and in general chemical gas sensors are semiconducting metal oxides (MOX) with a wide band gap [3]. The most commonly used MOX for this purpose are: SnO_2_ [4], ZnO [5,6], TiO_2_ [7,8], V_2_O_5_ [9], WO_3_ [10] and others. Also, some approaches use conducting polymers, phthalocyanines and graft copolymers of such materials in studies of gas sensing materials [11,12,13].

Recently, a lot of attention has been focused on the nanostructures of MOXs. The application of nanostructures and nanoporous films leads to a decrease in the operating temperature of the sensors and improves their sensitivity, response time and selectivity [14]. Nanostructures can have different morphologies, for example: nanotubes, nanorods, nanolumps, nanopowders or nanoflowers are obtained by various methods [15]. These methods include: controlled precipitation [16], sol-gel methodology [17,18], hydrothermal method [5,19,20], chemical vapor deposition (CVD), physical vapor deposition (PVD), electrospinning and the laser ablation method [21,22,23]. The modifications of the gas receptors based on ZnO nanostructures by the coverage of ZnO nanowires with a molecular sieve membrane were also proposed to improve sensor selectivity [24]. Many of these routes are inconvenient due to the complex and expensive synthesis procedure and expensive equipment required. 

MOX are used in resistive, gravimetric, optical, and other types of chemical sensors [25,26,27], with the most popular being resistive (conductometric) sensors [25,28]. A major problem is the reduction of their operating temperature, which is typically in the range of 200–700 °C [29,30]. In recent years, attempts have been made to solve this problem using UV radiation [30,31,32]. The application of UV allows for the gas detection at room temperature (RT), but response times under these conditions are often unsatisfactorily long [30,32,33]. There have only been a few studies discussing the combination of the UV illumination of the sensor at higher temperature and the mechanisms responsible for operation under such conditions [34,35]. One of the most important problems with MOX-based gas sensors is baseline drift, which has to be reduced to obtain the long-term stability of the sensors [36]. 

ZnO is an *n*-type semiconductor with direct band gap (e.g., ≈3.3 eV) [37]. This material is examined because of its possible use for the production of high-speed electronic elements [38], for the manufacture of lasers and light-emitting diodes of the emission of radiation in the ultraviolet range [39], but also in terms of its use as a waveguide layers and structures [40,41].

The paper discusses the resistive gas sensor based on ZnO nanostructures synthesized by the hydrothermal method. It is the continuation of our previous work [42], where the synthesis, characterization and gas-sensing properties of such materials was studied and discussed. This investigation showed that the sensors based on the ZnO nanostructures are significantly sensitive to NO_2_ at the temperature of 200 °C and at RT with UV activation.

In the current study, we focused on the combination of UV and temperature activation of the ZnO nanostructures to improve sensor dynamics, mainly a decrease in the response and recovery times and a reduction of baseline drift. The sensing mechanisms caused by temperature and UV activation of the sensor are also studied in this work.

## 2. Experimental

### 2.1. Sensor Fabrication

In the ZnO nanostructures synthesis processes, the following materials were used: Zn(NO_3_)_2_·6H_2_O (Sigma-Aldrich, Saint Louis, MO, USA), NaOH (Avantor Performance Materials, Gliwice, Poland), ethylenodiamine EDA (Sigma-Aldrich,) and ethanol (Sigma-Aldrich). ZnO was obtained by hydrothermal method described in detail in our previous work [42].

**Figure 1:**
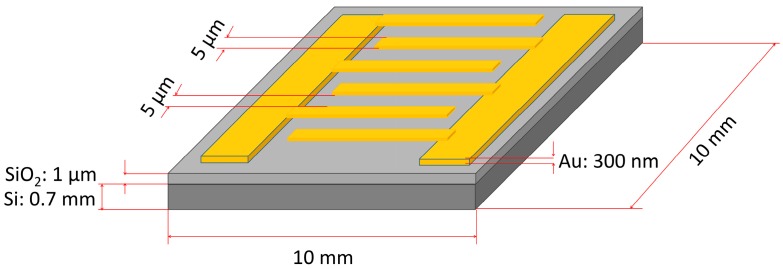
Scheme of inter-digital transducer (IDT) with dimensions. Figure not to scale.

ZnO nanostructures, which are the receptor material, were applied to the interdigital transducers (IDT) using the drop coating method. The IDT were made by the UV photolithography method from the gold film (300 nm Au on 2 nm Cr adhesion layer) on a silicon substrate with a layer of SiO_2_. Transducer dimensions are shown in the scheme presented in Figure 1.

The ZnO nanostructures were dispersed in pure hexane (POCH, Gliwice, Poland) using ultrasonic stirring for 5 min. After that, the suspension was dropped on IDT and dried at RT. The material which was not adhering to the transducer was removed with compressed air at a pressure of 5 bar. Prepared sensors were preheated at a temperature of 200 °C in ambient conditions for 30 min.

Four sensors were made and simultaneously tested; the results obtained for each of them were comparable. Results presented in this paper were obtained for the same sensors.

### 2.2. Gas Measurements Details

**Figure 2:**
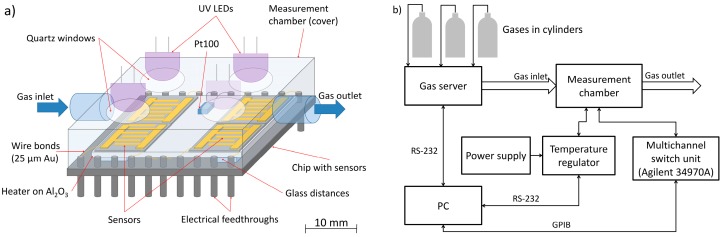
(**a**) Scheme of the measurement chamber; (**b**) Scheme of the measurement stand for gas sensors tests.

The tests of ZnO nanostructures were carried out at different gaseous atmospheres and under various environmental conditions such as temperature and electromagnetic (UV) illumination. The scheme of the measurement chamber consisting of the chip with four sensors and the cover is shown in Figure 2a. For the control of temperature, the measurement chamber is equipped with a thick film heater on the Al_2_O_3_ substrate and Pt100 temperature sensor. The cover has quartz windows and UV light emitting diodes (LEDs, λ = 390 nm), which provide the ability to illuminate the sample by UV radiation. Sensors are placed on the heater and the electrical contacts between the chip feed-throughs and sensor electrodes were made by gold wire with a diameter of 25 μm, using the ultrasonic wire-bonding technique (wire bonder 53XX-BDA, F&K DELVOTEC, Braunau, Austria). The chamber elements were made of chemical-resistant materials such as polytetrafluoroethylene (PTFE) and stainless steel.

A scheme of the measurement stand for gas tests is presented in Figure 2b. Data acquisition and measurement control was performed with the personal computer PC with LabVIEW software. The temperature was controlled and measured using a proportional-integral-derivative (PID) controller SR94 (Shimaden, Tokyo, Japan). In all cases, resistance measurements were carried out using a multi-switch unit 34970A (Agilent, Santa Clara, CA, USA) within the range of 10 MΩ, where the current source of 100 nA is used. 

The preparation of gas mixtures was provided by gas server based on mass flow controllers. The server enables the accurate production of a gaseous atmosphere consisting of any carrier gas and up to five different gases with precisely defined concentrations. 

During the measurements, two carrier gases, nitrogen 6.0 and synthetic air, were used and the flow of gas through the measuring chamber was keep at a constant level of 500 mL/min. Both carrier gases had similar relative humidity RH = 6 ± 1% at 23 °C. In the experiments, a standard mixture of NO_2_ 100 ppm in N_2_ was used. Measurements of the response of the ZnO structure to the gases consisted of alternating the addition of pure carrier gas and NO_2_ at a given concentration to the measuring chamber. In each cycle, the given gas concentration was increased. Measurements were carried out at RT = 23 °C and increased temperatures of 100 °C and 200 °C in dark conditions and using the UV activation.

### 2.3. Characterisation Methods

After the syntheses, the obtained structures were depicted by means of a field emission scanning electron microscope (FE-SEM) SUPRA 35 (ZEISS, Jena, Germany). The crystalline ZnO nanostructures were analyzed by X-ray diffraction (XRD) using the X’Pert Pro MPD diffractometer (PANalytical, Almelo, The Netherlands) with Cu anode (Cu Kα line—λ = 0.179 nm).

Raman investigations were performed on the Ntegra Spectra system (NT-MDT, Moscow, Russia) equipped with a charge-coupled device (CCD) detector (made by Andor, Belfast, UK) using green (532 nm) laser excitation. 

The morphology and distribution of the nanostructures on the transducer were investigated using scanning electron microscopy (Inspect S50, FEI, Hillsboro, OR, USA).

### 2.4. Temperature Measurements Details

Temperature measurements were carried out to study the relationship of the resistance of ZnO nanostructures and the temperature R(*T*) in different gaseous atmospheres and to examine the desorption of NO_2_. Before each series of tests, the sample was heated up in the given carrier gas (air or N_2_) to a temperature of 300 °C, then cooled to RT and exposed to the impact of 20 ppm of NO_2_ at RT for 30 min. In each of the atmospheres, the sample was heated, cooled and reheated. The temperature was varied in the range from 30 to 300 °C increased each time by 10 °C until stabilization of the resistance (time interval was approximately Δt = 2 min).

To determine whether the chemical transformations that occur during the heating of ZnO are endo- or exo-energetic, a differential scanning calorimetry (DSC) measurements were performed. The study was conducted using DSC 822e/500 (made by METTLER TOLEDO, Columbus, OH, USA) in a nitrogen atmosphere. Three different samples (each 5 mg of the nanostructures) were tested; each was exposed to different gaseous atmosphere before testing. Sequentially: sample 1 was heated to 300 °C in nitrogen, sample 2 was heated to 300 °C in air and sample 3 was heated to 300 °C in nitrogen, and, after cooling, treated with 20 ppm of NO_2_ for 30 min.

### 2.5. Spectral Measurements Details

In order to select the optical excitation wavelength, spectral studies of the absorption of electromagnetic radiation of the UV-Vis range were conducted. The measurements of the dependence of the sensors resistance and the excitation wavelength were performed.

The sensor was illuminated with light in the wavelength range from 300 nm to 750 nm when its resistance was monitored. The light source was a xenon lamp and a monochromator DH-10 (Jobin YVON, Lonjumeau, France) was used to tune the excitation wavelength. The wavelength was changed at a fixed time interval Δt = 1 min, increasing each time by 10 nm in the range of 400–750 nm and 5 nm in the range of 400–300 nm. The measurement was carried out in an atmosphere of atmospheric air in an open chamber at RT. Light intensity was kept at a constant level. The light intensity for the given wavelength and the LED spectrum were measured with a HR2000 SR+ spectrometer (Ocean Optics, Dunedin, FL, USA).

After selecting the optimum light wavelength and its source (UV LED), the reaction of the resistive structure to the UV light was measured. The measurement was based on testing the resistance in time, in dark conditions and in the exposure of the structure to the UV light. The measurement was conducted at RT at a constant flow of dry synthetic air.

## 3. Results and Discussion

### 3.1. Characterisation of ZnO Nanostructures

**Figure 3:**
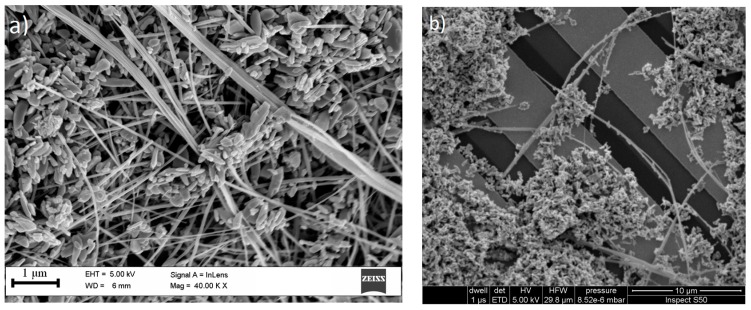
SEM images of: (**a**) ZnO nanostructures; (**b**) distribution of ZnO nanostructures on IDT.

Morphology of obtained with ZnO structures is presented in FE-SEM images in Figure 3a. ZnO nanostructures constitute a rich mixture of different geometrical forms, from nanobars and nanotubes with a predominantly size even a row of few μm, through nanotubes, nanobars up to nanograins with sizes up to single nm. 

The XRD measurement results were complementary to results presented in our previous paper [42]. The resulting diffraction pattern is characteristic of the structure of hexagonal ZnO (wurtzite). Wurtzite structure belongs to spatial group of P63mc (conformity tested in the card code ICSD No. 180052 “ZincOxide-Nanoparticles”). In this spectrum, there was no additional peaks. 

Recorded Raman spectra showed characteristic ZnO wurtzite modes at 334, 380, 438 and 579 cm^−1^ which are assigned as 2E2, E1TO, E2 and E1LO modes, respectively. Raman spectrum and it analysis was presented in detail in our previous paper [42]. Raman spectra did not show any changes after annealing to the 300 °C and after gas sensing experiments.

The applied sensor layers have good adhesion to the substrates (it does not detach from the transducer when exposed to fast gas flow and mechanical shocks) and a relatively uniform distribution of grains of ZnO nanostructures on the transducer. An example of the grain distribution can be observed in the SEM image in Figure 3b.

### 3.2. Temperature Effect of NO_2_ Sensing Properties of the Sensor in Different Carrier Gases

**Figure 4:**
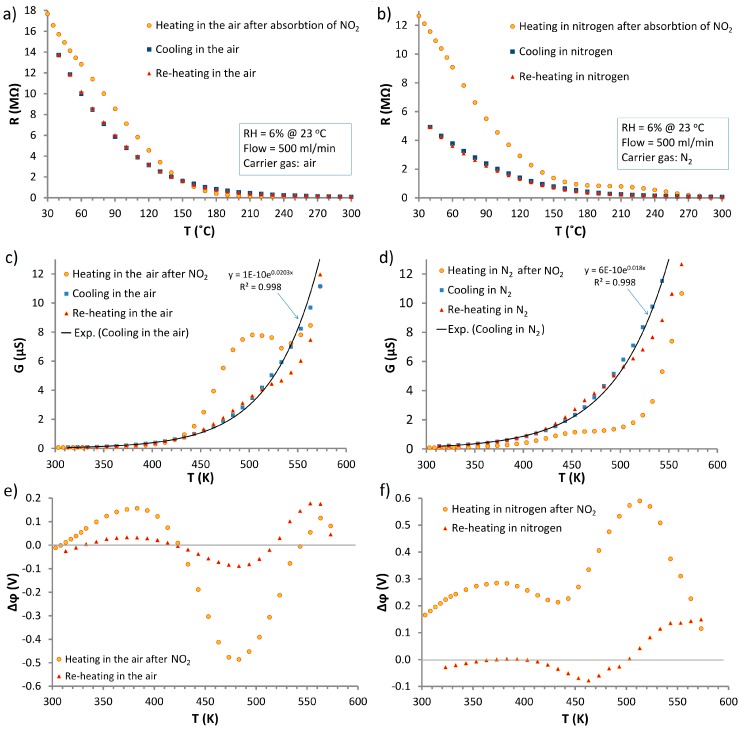
Temperature dependences of: (**a**,**b**) resistance; (**c**,**d**) conductance; (**e**,**f**) surface potential; for ZnO nanostructures saturated by NO_2_ at RT and then heating, cooling and re-heating in air and nitrogen.

Figure 4 shows thermal changes of resistance of the ZnO nanostructures on temperature for both tested carrier gases. From graphs R(*T*) (Figure 4a,b), it can be observed that the heating characteristics of the sample after interaction with NO_2_ are different from the cooling and reheating characteristics without contact with the analyte. In both air and nitrogen at temperatures in the range 30–180 °C, the resistance value after interaction with NO_2_ is higher than the resistance for the cooling and re-heating (Figure 4a,b). In this area, a derogation from the exponential function can also be observed. To further emphasize that at higher temperatures (180–300 °C) are noticeable difference data are expressed as a function of the conductivity to the temperature where G(*T*) = R^−1^(*T*). Characteristics G(*T*) where the temperature is expressed in Kelvins is shown at Figure 4c,d.

At higher temperatures after the interaction with NO_2_, different directions of derogations from the exponential characteristics (observed in cooling processes) were noted in air (Figure 4c) and nitrogen (Figure 4d). In the case of nitrogen at the temperature range 450–573 K, the conductivity after contact with NO_2_ is lower than in the case of cooling and reheating in clean carrier gas (Figure 4d). In contrast to other cases, in the air after interaction with NO_2_ at the temperature range 440–540 K, local maximum occurred. There are also some differences in the processes of cooling and reheating at temperatures higher than 520 K. 

In order to consider changes in the electrical parameters of ZnO nanostructures the values of changes in the surface potential sample Δϕ(*T*) were estimated. As the reference value, cooling characteristics in both carrier gases were used. These data were fit to exponential function (Figure 4c,d) using the least squares method. Acquired characteristics are in accordance with Equation (1), on the assumption that the surface potential depends on the temperature [43,44]:

(1) $${G=G}_{0}\exp\left(\frac{e\phi_{s}}{kT}\right)$$

where: G is conductance, ϕ_s_—surface potential, G_0_—parameter independent from *T* and ϕ_s_, k—Boltzman constant. 

To calculate the relative change in the surface potential Δϕ(*T*), the conductivity for the given case G_x_ was divided by the fitted conductivity in the carrier gas G_car_:

(2) $$\frac{G_{x}\left(T\right)}{G_{\mathrm{car}}\left(T\right)}=\frac{G_{0}\cdot \exp\left(-\frac{\phi_{x}(T)e}{kT}\right)}{G_{0}\cdot \exp\left(-\frac{\phi_{\mathrm{car}}(T)e}{kT}\right)}=\exp\left[-\frac{e}{kT}\left(\phi_{x}(T)-\phi_{\mathrm{car}}(T)\right)\right]=\exp\left(-\frac{∆\phi (T)e}{kT}\right)$$

Δϕ(*T*) is the difference between the potential in the case of heating the structure (after the interaction with NO_2_ and in a clean carrier gas) and the reference potential (cooling). From Equation (3), the relationship of changes in surface potential and temperature was determined:

(3) $$∆\phi \left(T\right)=-\ln\left(\frac{G_{x}(T)}{G_{\mathrm{car}}(T)}\right)\frac{kT}{e}$$

The characteristics of Δϕ(*T*) are shown in Figure 4e,f, where local extremes are observed. These extremes should correspond with the physicochemical changes taking place on the surface of ZnO nanostructures during heating. 

After interaction of the structure with NO_2_ in nitrogen, the Δϕ(*T*) within the whole range of tested temperatures was offset about 0.2 V (Figure 4e), which corresponds to an adsorption energy of NO_2_ on ZnO [45]. This is due to the oxidizing nature of NO_2_ that results in the accumulation of negative charge in the surface states. After heating in nitrogen, active centers are vacant, which results in differences between observed potential changes. This trend is not observed in the air (Figure 4e) where, at low temperatures, Δϕ is close to 0 V. This is due to the presence of oxygen, which occupies active centers left by NO_2_.

**Figure 5:**
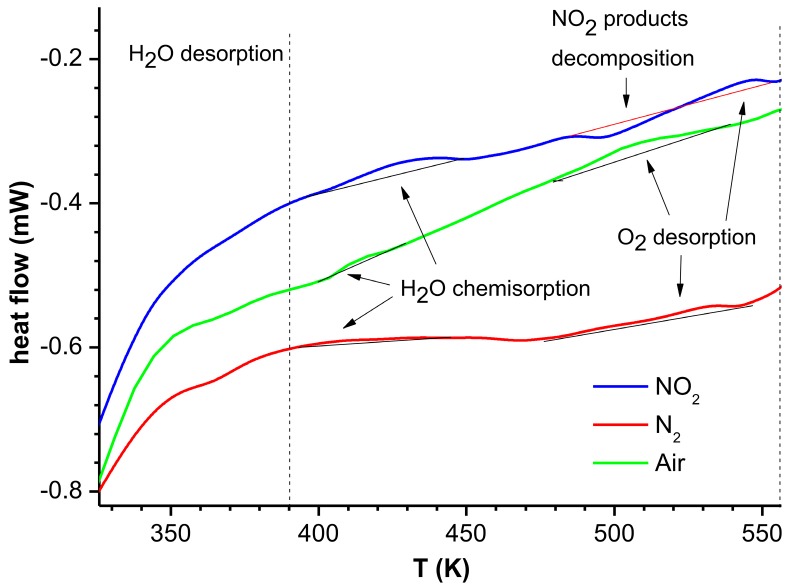
Results of DSC analysis for the heating of ZnO nanostructures heated to 300 °C in nitrogen (**red line**), air (**green line**) and after the contact with NO_2_ at RT (**blue line**).

DSC studies were conducted for the heating (Figure 5) and cooling of samples. In the cooling process, no changes in the thermal conductivity were observed, which confirms that the characteristics for cooling form a proper reference for calculating Δϕ(*T*).

In all the observed characteristics of Δϕ(*T*), the existence of a local maximum in the temperature range 320–400 K can be observed. At this temperature, physically adsorbed water (from the carrier gas RH = 6%) is removed from the structure. In all the DSC results for heating in the same temperature range, exothermic reaction of the water evaporation (Equation (4)) is observed (Figure 5):

(4) $$H_{2}O_{\left(\mathrm{ads}\right)}\to H_{2}O_{\mathrm{gas}}↑$$

At the temperature range 400–500 K, as a result of the catalytic decomposition [46] of water (remained in the sensor and provided in the carrier gas), its chemical adsorption occurs due to the dissociation into the OH^−^ group and proton (H^+^), which takes place according to the reaction (Equation (5)):

(5) $$H_{2}O_{\left(\mathrm{ads}\right)}\to {\mathrm{OH}}_{\left(\mathrm{ads}\right)}^{-}{\;+\;H}_{\left(\mathrm{ads}\right)}^{+}$$

The OH^−^ group is located on a surface lattice of zinc, and the proton is intercepted by the O^−^ ion and OH^−^ group is formed (Equation (6)).

(6) $$H_{\left(\mathrm{ads}\right)}^{+}{\;+\;O}_{\left(\mathrm{ZnO}\;\mathrm{surface}\;\mathrm{lattice}\right)}^{2-}↔{\mathrm{OH}}_{\left(\mathrm{ads}\right)}^{-}$$

In this temperature range, a decrease in Δϕ(*T*), are observed (Figure 4e,f). This is in accordance with the DSC, in which endothermic signal is observed in the same temperature range.

After the neutralization of OH^−^ groups (namely, the electron transmission to the conduction band of the semiconductor), hydroxyl groups can desorb as radials. Therefore, the chemical adsorption of one molecule of H_2_O causes the emergence of two OH groups, and a negative charge from O^2−^ is neutralized [47]. This leads to a decrease in Δϕ, providing electrons to the conduction band and creating two oxygen vacancies (Equation (7)).

(7) $${2\mathrm{OH}}_{\left(\mathrm{ads}\right)}^{-}\to {2\mathrm{OH}}_{\left(\mathrm{gas}\right)}^{\cdot }{\;+\;2e}^{-}$$

For NO_2_ adsorption on ZnO at RT and under nitrogen or air flowing condition, it is as follows [8,48]:

(8) $${2\mathrm{NO}}_{2(\mathrm{ads})}↔{\mathrm{NO}}_{3}^{-}{\;+\;\mathrm{NO}}_{(\mathrm{ads})\text{'}}^{+}$$

(9) $${\mathrm{NO}}_{\left(\mathrm{ads}\right)}^{+}{\;+\;O}_{\left(\mathrm{ZnO}\;\mathrm{surface}\;\mathrm{lattice}\right)}^{2-}↔{\mathrm{NO}}_{2(\mathrm{ads})\text{'}}^{-}$$

(10) $${\mathrm{NO}}_{2(\mathrm{ads})}^{-}{\;+\;\mathrm{NO}}_{2(\mathrm{ads})}↔{\mathrm{NO}}_{3(\mathrm{ads})}^{-}{\;+\;\mathrm{NO}}_{\left(g\right)}$$

Summary:

(11) $${3\mathrm{NO}}_{2(\mathrm{ads})}{\;+\;O}_{\left(\mathrm{ZnO}\;\mathrm{surface}\;\mathrm{lattice}\right)}^{2-}↔{2\mathrm{NO}}_{3(\mathrm{ads})}^{-}{\;+\;\mathrm{NO}}_{\left(g\right)}$$

**Figure 6:**
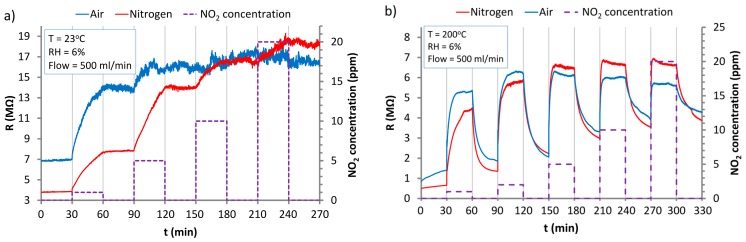
The response of the sensor based on ZnO nanostructures to the action of NO_2_ in the atmosphere of synthetic air and nitrogen at: (**a**) room temperature (RT); (**b**) at 200 °C.

Thus, the tested structure comprises of adsorbed NO_2_ in the form of NO_3_^−^. At RT, the desorption of NO_3_^−^ is not observed, as shown by the result presented in Figure 6a. At higher temperatures (450–550 K) decomposition of NO_3_^−^ to gaseous NO_2_ and O^−^_(ads)_ occurred:

(12) $${\mathrm{NO}}_{3(\mathrm{ads})}^{-}\overset{∆}{\to }O_{\left(\mathrm{ads}\right)}^{-}{\;+\;\mathrm{NO}}_{2\left(g\right)}$$

In the Δϕ(*T*) in the oxygen-free atmosphere (nitrogen—Figure 4f), after the interaction with NO_2_ in a temperature range 450–550 K, high maximum is observed. This is the effect of immediate adsorption of NO_2_ released in the reaction (Equation (12)), in accordance with reactions (8)–(10), which generates additional charges until NO_3_^−^ is exhausted. After that, a drop in Δϕ(*T*), which results from the thermal desorption of the remaining O^−^_ads_, is recorded. These processes are confirmed by the DSC analysis (Figure 5).

In the atmosphere of air Δϕ(*T*) (Figure 4e), in the temperature range 430–550 K, the deep minimum is observed (the situation is reversed in the case of N_2_). In this case, the only difference is the presence of a high concentration of oxygen in the carrier gas which efficiently occupies all active centers (released during the mentioned desorption processes). This hinders the resorption of NO_2_ released in Equation (12), causing its rapid evacuation from the structure. A high concentration of oxygen adsorbed on the active centers gives high efficiency of the chemisorption process of water (Equation (7)). As a result of this reaction, oxygen vacancies, which may be filled by oxygen from the carrier gas, are generated efficiently. If the speed of the generation of oxygen vacancies is higher than the speed of the adsorption of oxygen, the surface potential is lowered. Above 490 K, an increase in Δϕ(*T*) is observed due to oxygen desorption from the ZnO surface.

The above considerations have shown that the optimum temperature for the measurement of NO_2_ in the atmosphere of air should be around 200 °C.

### 3.3. Sensor Response to NO_2_ at Different Temperatures

The characteristics shown in Figure 6 prove that the ZnO nanostructures interact with the molecules of NO_2_ at RT and higher temperatures (200 °C) increasing their resistance. At RT (Figure 6a), after the removal of NO_2_ from the carrier gas, the desorption process is not observed; resistance of the structure is not reduced. This confirms that, at RT, a stable chemisorption of NO_2_ on ZnO nanostructures according to the reactions (Equations (8)–(10)) is observed. As a result, saturation of the structure and a lack of response to subsequent NO_2_ doses are observed. In the atmosphere of nitrogen, the resistance of the sensor under the influence of NO_2_ is increased more slowly than in air. This is caused by the increased amount of oxygen vacancies of ZnO nanostructures in an oxygen-free atmosphere. The saturation in both carrier gases occurs at a comparable level of resistance. This stems from the fact that NO_2_ and oxygen are absorbed in the same active sites.

At a temperature of 200 °C (Figure 6b), sensor response to NO_2_ is ca. 5 times higher than at RT.

At 200 °C, NO_2_ desorption takes place according to reaction (Equation (12)). This is consistent with the conclusions of Δϕ(*T*) and DSC measurements outlined above. 

It has to be observed that despite high responses, response and regeneration times of the sensor at 200 °C are unsatisfied. What is more, sensor is not scalable due to its significant baseline drift, especially in air.

**Figure 7:**
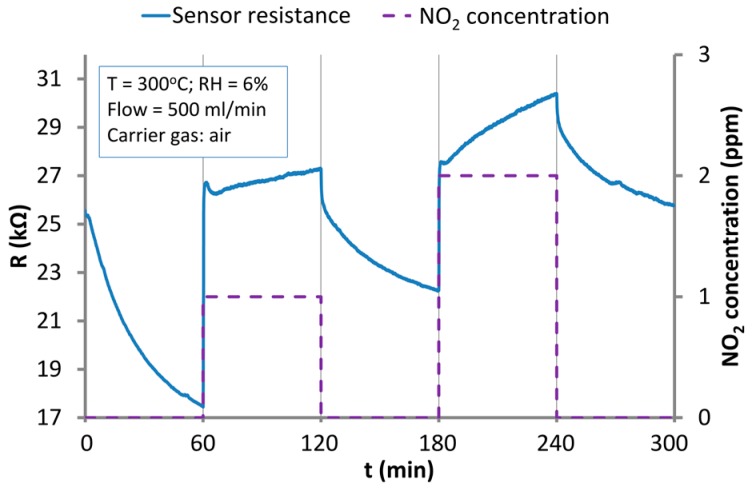
The response of the sensor based on ZnO nanostructures to the action of NO_2_ in the atmosphere of synthetic air at 300 °C.

An increase of the operation temperature to 300 °C (Figure 7) shortened the response time and also reduced the response value. What is more, baseline drift is still significant at this temperature even after 60 min cycles. 

The results presented above showed that elevated temperature does not cause full sensor recovery and significant senor baseline drift is observed, precluding the sensor from proper operation. To eliminate the above-mentioned problems, investigation of the influence of electromagnetic radiation to the operating sensor was conducted.

### 3.4. Influence of UV-Vis Irradiation on the Sensors

**Figure 8:**
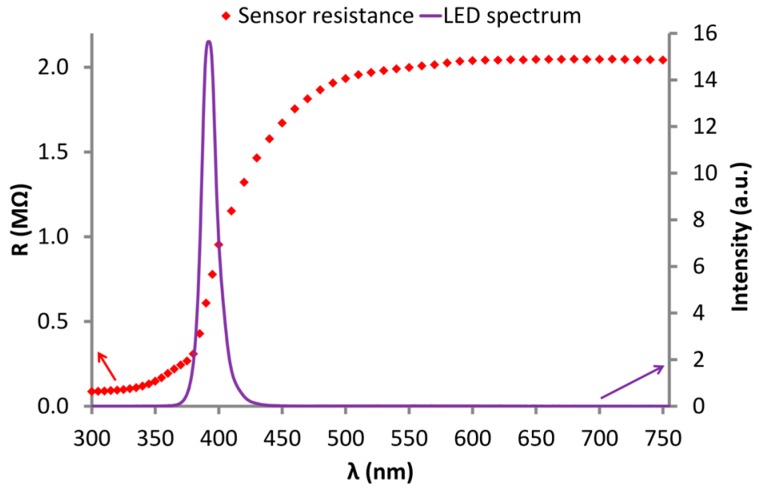
The relationship of the resistance of the ZnO nanostructures on the wavelength R(λ) (at ambient conditions) and the UV LED spectrum (violet line).

To estimate the optimal wavelength (λ) for sensor activation, spectral studies of the sensor resistance R(λ) were conducted (Figure 8). The resistance changes caused by light can be observed at wavelengths below 500 nm. The most rapid changes of resistance are observed in the range of 380–420 nm, whereas a decrease in the intensity of changes occurs between 380 and 390 nm, corresponding to the width of the ZnO band gap (3.2–3.3 eV) [49,50]. Spectral investigations show that optical excitation of the ZnO nanostructures near UV light (370–400 nm) is sufficient. As a UV source, LED (λ = 390 nm), the spectrum of which is shown in Figure 8, was selected. 

After sensor illumination by UV LED (2.0 ± 0.5 mW/cm^2^), rapid resistance decrease of the sensors (by three orders of magnitude) was observed. Such resistance drop is mainly a result of the increase in charge carriers caused by the photogeneration process (Equation (13)).

(13) $${h\nu =e}^{-}{\;+\;h}^{+}$$

The resistance also changes due to the photocatalytic phenomenon, which caused the adsorption and desorption of oxygen (on active centers) [30] according to (Equations (14) and (15)).

(14) $$e^{-}{\;+\;O}_{2(g)}\to O_{2(\mathrm{ads})}^{-}$$

(15) $$h^{+}{\;+\;O}_{2(\mathrm{ads})}^{-}\to O_{2(g)}$$

**Figure 9:**
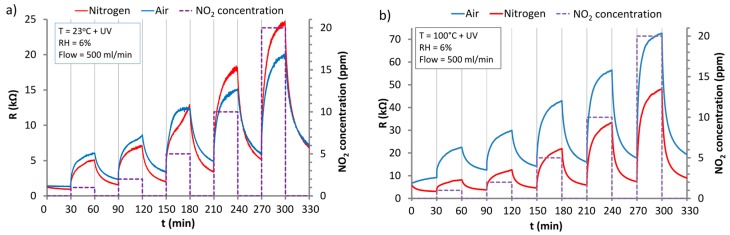
The response of the ZnO sensor to the action of NO_2_ under UV irradiation in the atmosphere of synthetic air and nitrogen at: (**a**) RT; (**b**) 100 °C.

Results of the sensor response to NO_2_ at RT under continues UV irradiation are shown in Figure 9a. Using the UV radiation made the sensor scalable, and reduced the baseline drift in comparison to higher operating temperatures.

Adsorption of NO_2_ under UV illumination at RT occurred according to [51,52]:

(16) $${2\mathrm{NO}}_{2\left(g\right)}{\;+\;e}_{\left(h\nu \right)}^{-}\to {2\mathrm{NO}}_{\left(g\right)}{\;+\;O}_{2(\mathrm{ads})}^{-}$$

(17) $${\mathrm{NO}}_{2(g)}{\;+\;e}_{\left(\mathrm{hv}\right)}^{-}\to {\mathrm{NO}}_{\left(g\right)}{\;+\;O}_{\left(\mathrm{ads}\right)}^{-}$$

Reactions (Equations (16) and (17)) show that, under UV conditions, products of ZnO interaction with NO_2_ are chemisorbed oxygen ions, which can desorb from the lattice according to Equation (15).

The described results proved that the obtained sensor can be used to measure low NO_2_ concentrations at RT. However, the remaining baseline drift, long response and recovery times made the sensor inconvenient for many applications. This is the reason why measurements with the combination of thermal and UV activation were made. 

**Figure 10:**
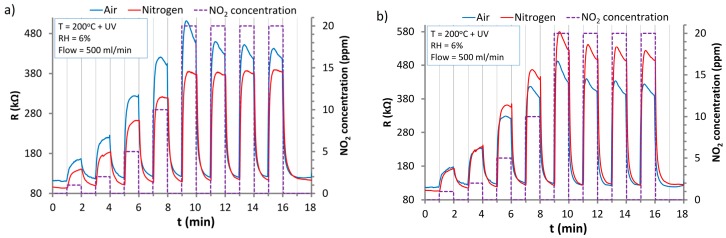
The responses of the ZnO sensors to the action of NO_2_ under UV irradiation in the atmosphere of synthetic air and nitrogen at 200 °C: (**a**) Sensor 1; (**b**) Sensor 2.

The sensor reaction to NO_2_ at 100 °C with UV activation is presented in Figure 9b. These results showed that the baseline drift is reduced but it is still significant. Rewarding results were obtained at 200 °C with UV activation (Figure 10).

Responses obtained for two chosen sensors tested simultaneously at 200 °C and under UV irradiation are presented in Figure 10. In this case, sensor response and recovery times are significantly shorter than in the above-mentioned cases (Figure 6 and Figure 9); even in a 1 min cycle, full response and recovery of the sensors can be observed. What is more, the baseline drift is reduced to a negligible level. This is evidence that in such conditions, fast and repeatable desorption of NO_2_ and its decomposition products from the ZnO surface were according to Equations (12) and (15).

For two sensors with extreme values of the base resistances (Figure 10), the values were on the level of 95–108 kΩ in the nitrogen and 112–116 kΩ in air. These resistances are higher than in the case of UV activation at RT and 100 °C (Figure 9). This is the result of the thermal recombination of the photogenerated charge carriers. At 200 °C, the equilibrium state between photogeneration and recombination of carriers is reached at different levels than at lower temperatures.

To make quantitative comparison of the sensors parameters at different conditions response times (t_resp90%_), regeneration times (t_reg90%_) and responses (calculated using Equation (18)) of the sensors were calculated.

(18) $$\mathrm{Response}=\frac{R_{g}{\;-\;R}_{a}}{R_{a}}\cdot 100\%$$

where R_a_ is the sensor resistance in the carrier gas and R_g_ is the sensor resistance after the action with NO_2_. Average values of these parameters calculated for all four sensors are presented in Table 1, where deviations from average values were calculated as a difference between extreme values in the population. From Table 1, it is clearly visible that t_resp90%_ and t_reg90%_ at 200 °C with UV activation are significantly shorter than in other cases exceeding 300 °C, where t_resp90%_ is at a comparative level; however, at 300 °C, t_reg90%_ is unacceptably high. This shows that only at 200 °C with UV sensors are dynamics at an acceptable level. What’s more, deviations from the average time values are relatively small (≤8 s), proving that the sensors and the manufacturing process are repeatable. The improvement of sensor dynamics at 200 °C with UV is connected to the decrease of sensor response values in comparison with other analyzed cases (exceeding 300 °C). At 200 °C with UV the equilibrium state between adsorption and desorption rates of NO_2_ products is achieved faster due to higher desorption rate (combination of reactions Equations (12) and (15)). The results of this phenomena are much shorter response and recovery times but also the decrease in the response value. However, it should be stressed that the t_resp90%_, t_reg90%_ and responses of investigated sensors to 1 ppm of NO_2_ at 200 °C with UV are comparable or better than in other literature reports [42,53].

The investigations of ZnO nanostructure reactions to other gases (H_2_, NH_3_) and RH were presented in our previous work [42], where the structures showed selectivity to NO_2_ at all measured conditions.

**Figure 11:**
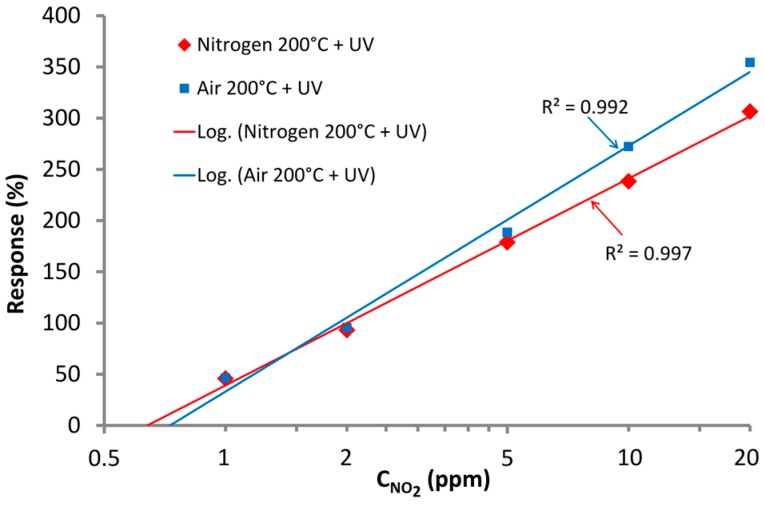
Representative sensor calibration curves for 200 °C with UV activation in air and nitrogen conditions.

It has to be mentioned that the presented sensors at optimal conditions (200 °C + UV) are fully scalable. In Figure 11, the calibration curves for representative sensor in semi-logarithmic scale are presented. In both cases (in air and nitrogen conditions), logarithmic functions were fitted to the data; these fittings show that sensor responses can be easily linearized with good approximations (R^2^ > 0.99). The estimated limit of detection of the sensors are at the level of ≈800 ppb in nitrogen and ≈700 ppb in air. 

## 4. Conclusions

In this paper, extensive studies of temperature and carrier gas influence on NO_2_ sensing properties of the ZnO nanostructures with discussion of the sensing mechanisms were conducted. The optimal operating temperature (200 °C) was estimated. The investigation of UV-Vis influence on the sensor resistance presented in this work showed that wavelengths in the range of 370–400 nm can be applied to photo-activation of the ZnO nanostructures. Thus, the inexpensive UV source (UV LED, λ = 390 nm) can be used for excitation of the sensor and activation of its photocatalytic properties. 

Investigations presented in this paper showed that the combination of temperature and UV activation of the sensors based on ZnO nanostructures can significantly improve sensor dynamics. The presented results prove that at 200 °C with UV activation, sensor responses and regeneration times both decreased. In comparison with the single activation method (temperature or UV), these times decreased from about 10 min (or more) to less than 40 s. What is more, the baseline drift was practically eliminated, even in comparison to the higher temperature of operation (300 °C). At the suggested optimal conditions, sensors operate very stably and are fully scalable. Suggested sensor manufacturing proses (drop coating) and the material synthesis (hydrothermal method) are simple and relatively inexpensive. The UV activation can be realized by standard or SMD (Surface Mounted Devices)-packed LEDs which are also inexpensive. Our study showed that sensor responses to NO_2_ and response/regeneration times are comparable from sensor to sensor in air and nitrogen conditions, which suggests that the proposed simple technology connected with well-chosen operation conditions is repeatable. The operating temperature is comparable with other similar approaches, however the energy consumption can be limited through miniaturization of the transducers, for example using MEMS (microelectromechanical system) technology [54,55].

The combination of thermal and electromagnetic activation of the sensors based on MOX semiconductors can improve their metrological properties. However, it has to be observed that sensor responses decrease under the proposed conditions. Thus, the compromise between good sensor dynamics and the value of sensor responses has to be made.

## Figures and Tables

**Table 1:** Summary of average values of sensors: response times (t_resp90%_), regeneration times (t_reg90%_) and responses to 1 ppm of NO_2_ at different conditions.

Conditions	Carrier Gas	t_resp90%_ (s)	t_reg90%_ (s)	Response (%)
200 °C + UV	Air	34 ± 6	33 ± 3	49 ± 4
	N_2_	35 ± 8	34 ± 1	54 ± 17
100 °C + UV	Air	1085 ± 90	975 ± 230	157 ± 21
	N_2_	840 ± 500	965 ± 10	147 ± 23
RT + UV	Air	1030 ± 220	1115 ± 10	304 ± 74
	N_2_	1040 ± 120	1032 ± 140	494 ± 125
200 °C	Air	577 ± 262	573 ± 386	257 ± 50
	N_2_	1043 ± 70	652 ± 96	589 ± 25
300 °C	Air	25 ± 2	>1800	38 ± 30
